# EUS assisted transmural cholecystogastrostomy fistula creation as a bridge for endoscopic internal gallbladder therapy using a novel fully covered metal stent

**DOI:** 10.1186/1471-230X-14-164

**Published:** 2014-09-23

**Authors:** Nan Ge, Zhiguo Wang, Siyu Sun, Sheng Wang, Guoxin Wang, Shiwei Sun, Linlin Feng, Fei Yang, Wenzhuang Ma, Shupeng Wang, Xiang Liu, Jintao Guo, Wen Liu

**Affiliations:** Endoscopy center, Shengjing Hospital, Shenyang, China; Endoscopy center, Zhongxin Hospital, Taipei, Taiwan; Anesthesiology department, Shengjing Hospital, Shenyang, China; The Shengjing Hospital, China Medical University, No. 36, Sanhao Street, Shenyang, Liaoning Province China 110004

**Keywords:** Endoscopic-ultrasound, Cholecystectomy, Mental stent

## Abstract

**Background:**

Laparoscopic cholecystectomy (LC) has become the “gold standard” for treating symptomatic gallstones. Innovative methods, such as a scarless therapeutic procedure through a natural orifice are being introduced, and include transgastric or transcolonic endoscopic cholecystectomy. However, before clinical implementation, instruments still need modification, and a more convenient treatment is still needed. The aim of this study was to evaluate the feasibility of endoscopic internal gallbladder therapy such as cholecystolithotomy in an animal survival model.

**Methods:**

Four pigs underwent endoscopic-ultrasound (EUS)-guided cholecystogastrostomy and the placement of a novel covered mental stent. Four weeks later the stents were removed and an endoscope was advanced into the gallbladder via the fistula, and cholecystolithotomy was performed. Two weeks later the pigs were sacrificed, and the healing of the fistulas was assessed.

**Results:**

EUS-guided cholecystogastrostomy with mental stent deployment was successfully performed in all the animals. Four weeks after the procedure, the fistulas had formed and all the stents were removed. Endoscopic cholecystolithotomy was performed through each fistula. All the animals survived until they were sacrificed 2 weeks later. The fistulas were found to be completely healed.

**Conclusions:**

This study reports the first endoscopic transmural cholecystolithotomy after placement of a novel mental stent in an animal survival model.

## Background

Laparoscopic cholecystectomy (LC) is considered to be the “gold standard” for the treatment of symptomatic gallstones [1, 2]. The field of natural orifice transluminal endoscopic surgery (NOTE) has recently been developed and is becoming popular because it provides faster recovery times and better cosmetic results [3–5]. To further improve NOTE surgery to treat gallstones, many technical shortcomings of the instruments need to be overcome. A more simple way to realize the transluminal endoscopic gallstones removal is needed.

The aim of our study using an animal survival model was to evaluate the feasibility of our newly developed transmural cholecystolithotomy through a fistula formed by a stent, which had been previously placed during an EUS-guided transmural cholecystogastrostomy.

## Methods

### Animals

The study was approved by the board of the China Medical University and has adhered to the ARRIVE guidelines [6]. Large white healthy pigs (n = 4, 30–40 kg) were fasted for 24 hours. During the surgical procedures, the pigs were administered general anesthesia with endotracheal intubation. No antibiotic drugs were given before the procedures. The pigs resumed a normal diet after the procedures.

### Stent

**Figure 1:**
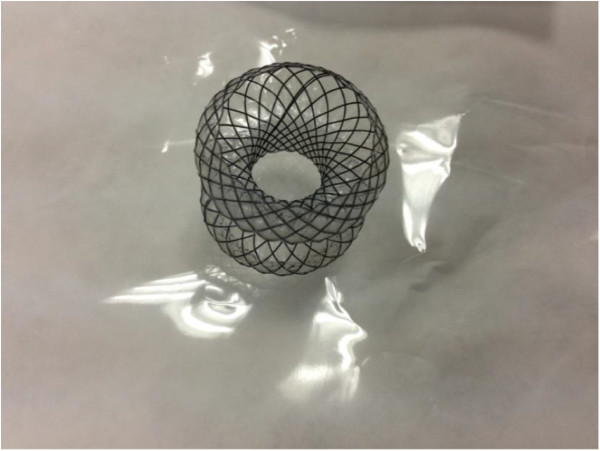
**The covered mental stent.**

The stent (Micro-Tech/Nan Jing CO., Ltd. China) had a self-expanding nitinol mesh design, with 2 large flared ends. Fully expanded, the 2 ends measured 20 mm in diameter. The waist of the stent measured 10 mm in diameter and the stent was 35 mm long. The 2 large flared ends were designed to protrude against the adjacent luminal walls with moderate pressure. The stent was fully covered by a polyester membrane, which prevents leakage and allows easily retrieval (Figure 1). The stent delivery system was compatible with the 3.8-mm working channel of the echoendoscope (Pentax EG-3830-UT, Tokyo, Japan).

### Cholecystogastrostomy

A longitudinal echoendoscope (Pentax EG-3830-UT) with a working channel of 3.8 mm was introduced to scan for the gall bladder and mark the puncture point. The contact zone (*i.e.*, the closest approximation of the region between the gastric wall and the gall bladder wall) was identified. Color Doppler then was used to identify interposing vessels and to avoid them during puncture. An EchoTip Ultra endoscopic ultrasound needle (19-G, Wilson-Cook Medic, USA) then was introduced via the working channel of the echoendoscope, and the gallbladder was punctured under EUS guidance. A sample was aspirated to confirm that the punctured structure was gallbladder. A guide wire (Tracer Metro Direct Wire Guide, 0.035 in/480 mm; Wilson Cook Medical Inc.) was left in the gallbladder with several loops, and the needle was removed. The needle path was then dialated by the cystotome(10-Fr, Wilson Cook Medic).

The stent (10 mm/35 mm; Micro-Tech/Nan Jing CO., Ltd.) was slowly deployed into the gallbladder under the guidance of EUS, until the distal flared end was completely open. Gentle traction was applied to pull the gallbladder wall close to the gastric wall. Then, under endoscopic surveillance, the remainder of the stent was deployed, keeping the proximal end in sight. The stomach was irrigated with saline, and EUS scanning was used to confirm the position of the stent and rule out further leakage.

### Transgastric cholecystolithotomy

The animals were monitored daily for signs of peritonitis. 4 weeks after the cholecystogastrostomy, the pigs were anesthetized and examined using a standard gastrointestinal endoscope. If the stent remained in place, it was removed using a foreign-body forceps or snare. The endoscope was advanced into the gallbladder via the fistula formed by the stent. Any foreign body deposited in the gallbladder could be removed by the stone basket, which was used to simulate the process of stone removal.

### Postoperative care

Finally, the pigs were sacrificed 2 week after this procedure. The peritoneal cavity was studied for evidence of adjacent organ injury, bleeding, or gross peritonitis.

## Results

**Figure 2:**
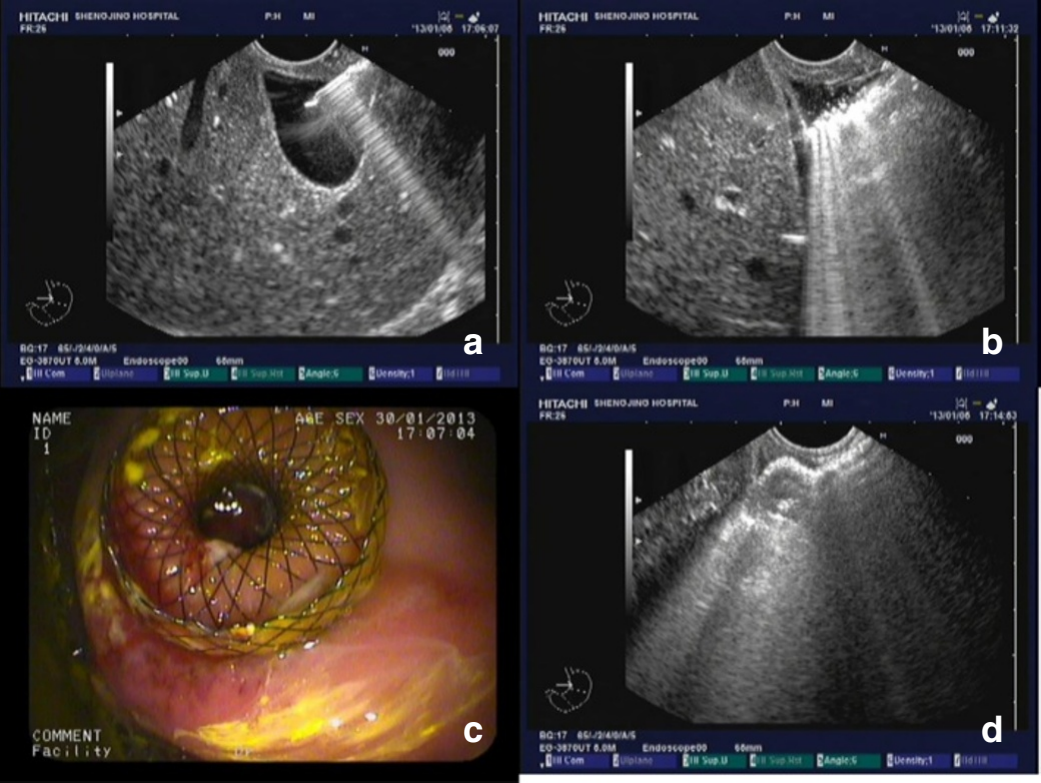
**Cholecystogastrostomy. a**. Endoscopy needle piercing the gallbladder. **b**. Dilation of the needle path using the cystotome, with a large amount of bile leakage. **c**. Endoscopic view of the deployed stent. **d**. Endoscopic ultrasound image of the deployed stent.

Both the cholecystogastrostomy and transgastric cholecystolithotomy were successfully performed in the 4 animals. The sites for fine needle puncture were the gastric antrum in 3 pigs and the duodenal bulb in 1. In 3 pigs, the gallbladder was near the gastrointestinal tract (GI), allowing a satisfactory puncture site. In the remaining pig, the gallbladder was located within 1 cm of the GI tract, and there was interposing hepatic tissue. During dilation of the needle tract, there was bile leakage from the gallbladder in all 4 animals. The metal stents were all successfully deployed (Figure 2). One day after cholecystogastrostomy, a single animal was slightly lethargic and anorexic, but resumed a normal diet the following day. None of the animals manifested signs of peritonitis.

**Figure 3:**
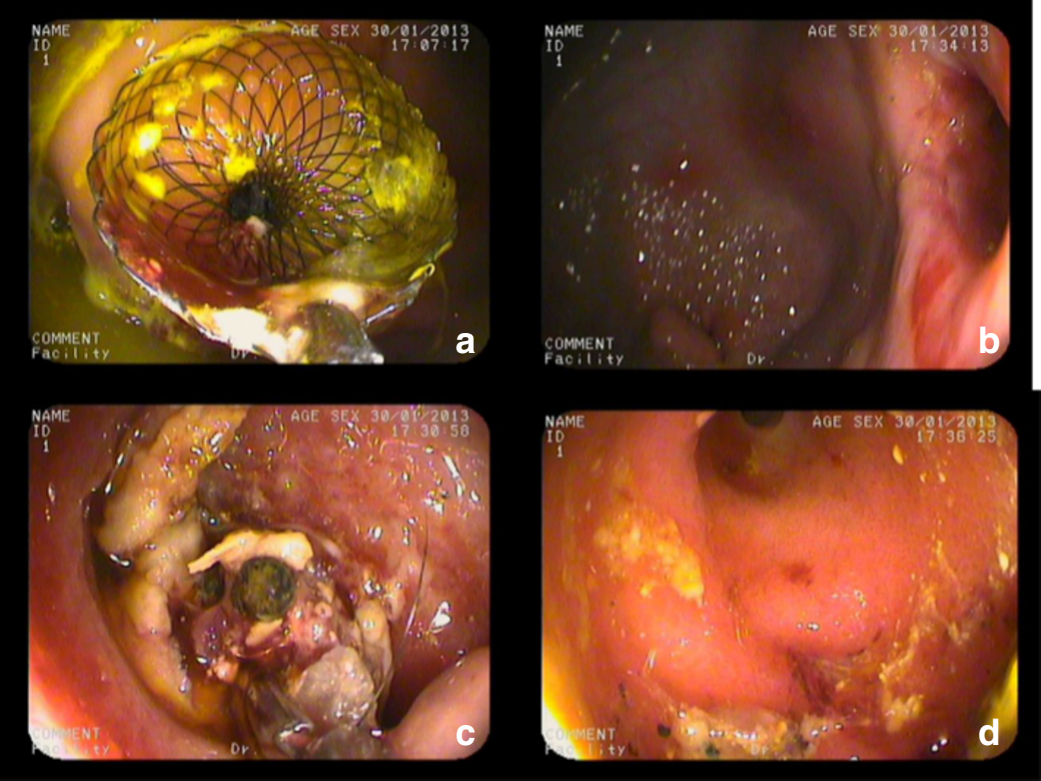
**Gastric transmural cholecystolithotomy. a**. Endoscopic view of the deployed stent. **b**. A fistula was formed after stent removal. **c**. Endoscopic view of the gallbladder cavity. **d**. After the gallbladder was emptied of contents, the neck was clearly viewed by endoscope.

Four weeks after the cholecystogastrostomy, the stents were found where they were originally placed in all the animals. All the stents were easily removed using the foreign body forceps and without much bleeding. Standard endoscopy revealed that the gallbladders were filled with food debris, which was removed in all animals using the stone basket (Figure 3). The endoscope allowed clear visualization of the gallbladder cavity, including the fundus and neck.

### Postoperative survival and necropsy

**Figure 4:**
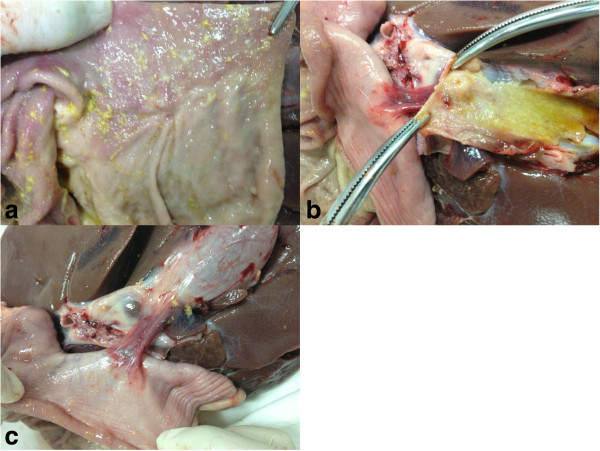
**Necropsy. a**. The stoma in the stomach was well healed 2 weeks after stent removal. **b**. The stoma in the gallbladder was well healed 2 weeks after stent removal. **c**. After the fistula was healed, the gastric and gallbladder walls were attached to each other by a short band of connective tissue.

All animals survived without complications after the transgastric cholecystolithotomy with simulated stone removal. They tolerated a regular diet within several hours after recovering from anesthesia. There were no clinically apparent adverse effects observed during the following week. Two weeks after the procedure, gastroscopy showed a white scar in the stomachs of all the animals. At necropsy, there was no evidence of organ injury, bleeding, or gross peritonitis. The incisions appeared well healed in both the stomach and gallbladder. There were no fistulas found in the stomachs of any of the animals. The gastric and gallbladder walls had adhered to each other by a short band of connective tissue formed by the healing fistula (Figure 4).

## Discussion

Acute cholecystitis and choledocholithiasis are common problems. Gallbladder surgery has been used to treat these conditions for almost 300 years. A major evolutionary step in gallbladder surgery was the change from laparotomy to laparoscopy. Although cholecystectomy remains the standard treatment for gallstones, it is a traumatic procedure and is associated with increased incidence of dyspepsia, calculus of the common bile duct, and colon carcinoma [7]. A novel advancement in treatment is to perform a scarless natural-orifice cholecystectomy. Treatment of a gallbladder polyp through a cholecistogastric fistula was first reported by Chen et al. [8], however the Cholecystoenterostomy may be a complication of gallstone disease rather than medical intervention. In 2004, Kalloo et al. first reported accessing the peritoneal cavity through a transgastric incision [9]. In 2006, Reina et al. reported performing a transcolonic endoscopic cholecystectomy [4]. However, these new techniques still have technical shortcomings with regard to instrument modification that must be overcome before their clinical application. Interventional EUS therapies are the unique and powerful newly developed clinical practice in treating many diseases [10–15]. For our study, we evaluated EUS-assisted transmural cholecystolithotomy performed after cholecystogastrostomy to place a stent without the use of fastening accessories such as T-anchor suturing [16]. It may not just the method to facilitate gallbladder drainage [17–20]. Technically, this may be an easy way to perform transmural cholecystogastrostomy to treat cholelithiasis.

EUS-guided cholecystogastrostomy with stent deployment offers several advantages over other cholecystolithotomy techniques currently in use. First, the covered metal stent that we used, with its 2 large flared ends, could more firmly hold the gastric wall against the gallbladder wall than other covered mental stents [21–23]. The shape of the flare end in this study was differently designed from the others [24, 25]. The flare ends were with blunt edge which would keep the mucosa from further injury. We did not observe additional leakage after the stents had deployed. The results from the first month demonstrated the efficiency of the anastomosis and that the procedure was well tolerated. This procedure could also be used for gallbladder decompression, with bile drainage into the GI tract.

A fistula had already formed by 4 weeks after the cholecystogastrostomy, providing a conduit from the gallbladder to the GI tract. The gastrointestinal endoscope easily entered the gallbladder, which could facilitate stone removal. In our experiment, the fistula was durable for many times enter until the deposit was completely removed for the gallbladder.

Because the endoscope was inside the gallbladder, it could clearly visualize the cavity, thus minimizing the possibility of leaving residual gallstones. Furthermore, this access might facilitate argon plasma coagulation and endoscopic mucosal resection to treat diseases involving gallbladder membranes. After the treatment, the fistula should heal, resulting in the possibility that gallbladder function could be preserved. Furthermore, EUS-guided cholecystolithotomy with stent deployment could be an option for elderly debilitated patients at high risk for surgery and for patients with cosmetic preferences.

Percutaneous cholecystolithotomy has been described as another alternative to laparoscopic cholecystectomy for high-risk patients and for those with adherent gallbladders that are not amenable to laparoscopic cholecystectomy [26]. However, the gastric transmural endoscopic approach satisfies the cosmetic demands of some patients and maintains bile circulation within the GI track, thus avoiding dyspepsia and electrolyte disturbances.

Potential limitations of this method include bilomas, hemorrhage, and infections, especially associated with dilation of the needle track intended for the fistula. In our study, there was bile leakage in all 4 animals when the needle pierced the gallbladder and during the dilation procedure. However, the leakage stopped after the stent was deployed, and the animals appeared to tolerate cholecystogastrostomy well, with no antibiotics needed prophylactically or for the duration of their survival. Our experience indicates that the amount of bile leakage did not require further intervention [27]. In the event of a large amount of bile leakage, advancement of the stent delivery system into an empty gallbladder might be difficult. Therefore several loops of guide wire should be coiled within the gallbladder after needle puncture to maintain the shape of the organ. This was the procedure widely accepted during the EUS-guided pancreatic pseudocyst drainage [28].

No hemorrhages were observed throughout the endoscopic process. Although we did not experience any difficulty during cholecystogastrostomy, it is possible that if the gallbladders had been located a little farther from the GI tract and if it were difficult locate an appropriate puncture site, the formation of an anastomosis might have been challenging.

There is a risk that the stent could be poorly positioned in the abdominal cavity during deployment; therefore EUS guidance is very important. In addition, caution should be employed when identifying appropriate patients for this procedure. Finally, a comparative study between this technique and other transmural procedures used for cholecystogastrostomy is needed. Improvement of the instruments used in this procedure also needs further study. Improvement would involve the integration of fistula dilation into the stent delivery system, because it would minimize leakage and prevent shifting of the gallbladder, since there would be no need to change instruments.

## Conclusion

This EUS-guided placement of a novel metal stent provided a safe and simple method for performing endoscopic cholecystogastrostomy, which can subsequently enable cholecystolithotomy and other procedures for treating biliary disease.
